# Impact of the COVID-19 Lockdown and Confinement Measures on the Musculoskeletal Health of the Urban Geriatric Population

**DOI:** 10.7759/cureus.19212

**Published:** 2021-11-02

**Authors:** Siddhartha Sharma, Riddhi Gohil, Sandeep Patel, Kamal Kishore, Amarjeet Singh, Rajesh K Rajnish, Mandeep S Dhillon

**Affiliations:** 1 Orthopaedics, Postgraduate Institute of Medical Education and Research Chandigarh, Chandigarh, IND; 2 Biostatistics, Postgraduate Institute of Medical Education and Research Chandigarh, Chandigarh, IND; 3 School of Public Health, Postgraduate Institute of Medical Education and Research Chandigarh, Chandigarh, IND

**Keywords:** confinement, elderly, geriatric, musculoskeletal disorders, lockdown, sars-cov-2, covid-19

## Abstract

Background

Owing to the coronavirus disease 2019 (COVID-19) pandemic, a nationwide lockdown was imposed in India, with strict confinement measures imposed on the elderly. Because mobility and regular physical activity are considered to be the key determinants of musculoskeletal health, this study aimed to investigate the effect of lockdown and confinement measures on the musculoskeletal health and activities of daily living of the urban geriatric population.

Methodology

A cross-sectional survey was conducted among the elderly aged ≥60 years. The survey instrument consisted of a questionnaire, a modified Nordic Musculoskeletal Questionnaire (mNMQ), and the Barthel activities of daily living (ADL) index. The net mNMQ score and Barthel ADL index were compared before and during the lockdown. Univariate and multivariate analyses were performed to determine which factors could result in the worsening of the net mNMQ score. In addition, floor and ceiling effects of the net mNMQ score were determined.

Results

In this study, a total of 105 out of 150 eligible participants were enrolled. A significant decline in physical activity status was noted during the lockdown. Overall, 54.3% of the respondents reported that their medical services were impacted during the lockdown. The net mNMQ score showed a significant worsening during the lockdown (P < 0.0001). A statistically significant increase in the modified NMQ score was noted for the lower limb (P < 0.0001) and spine (P = 0.002), but not for the upper limb (P = 0.052). Elderly whose medical services were impacted during lockdown had significantly worse net mNMQ scores than those whose services were not impacted (odds ratio = 6.16; 95% confidence interval = 2.51-15.08; P = 0.0001). Age, gender, ambulatory status, increase in body weight, and indulgence in exercise before and during lockdown had no effect on the change in the net mNMQ score. A significant ceiling effect was noted in the mNMQ score; however, no floor effect was noted.

Conclusions

Lockdown and confinement measures resulted in a significant decline in physical activity as well as the overall musculoskeletal health of the urban geriatric population in the present study. Hence, policymakers should ensure uninterrupted medical care to the elderly during extended periods of confinement and develop optimal home-based physical activity programs to counter the problems associated with sedentarism.

## Introduction

On March 11, 2020, the World Health Organization (WHO) declared coronavirus disease 2019 (COVID-19) as a pandemic [1]. After initial estimates pegged the reproduction number (R0) of the outbreak at higher than 1, a nationwide lockdown was announced by the Government of India on March 24, 2020. The unprecedented restrictions and confinement measures imposed by the lockdown affected people from all walks of life. However, the strictest confinement measures were enforced on the elderly because of the high morbidity and mortality of COVID-19 in this age group [2].

It is well-established that musculoskeletal disorders are a major cause of disability in the geriatric population [3]. The elderly living with these problems experience joint pain, stiffness, fatigue, and muscle weaknesses. Continued mobility and regular exercise are the key components of managing these disorders, both of which were affected by the lockdown and the consequent confinement [4]. In addition, several routine medical services such as outpatient consultations and elective orthopedic surgeries were suspended.

Hence, we conducted a community-based survey to determine the effects of lockdown and confinement measures on the musculoskeletal health of the geriatric population in an urban, north Indian city. We hypothesized that the lockdown would lead to a decline in the physical activity status of the elderly and a worsening of musculoskeletal problems.

## Materials and methods

Ethical considerations, study design, and sample selection

This study was conducted among the participants (aged >60 years) enrolled in an ongoing prospective cohort study that aims to assess the predictive factors for geriatric fractures. The study was approved by the Institutional Ethics Committee (IEC/PGI/2019/000738). Informed and written consent was obtained from all participants enrolled in the study. Additionally, telephonic consent was obtained from each participant for this survey. A telephone-based survey was conducted in accordance with the SUrvey Reporting GuidelinE (SURGE) [5].

Inclusion and exclusion criteria

Community-dwelling elderly aged >60 years who were able to provide accurate history; had no history of dementia, neurological, psychiatric, or cognitive disorders that can impair recall; and were willing to participate in the study were included. Elderly with impaired recall, dementia, cognitive disorders, and those unwilling to participate were excluded from the study.

The research tool

The research tool comprised three separate elements, viz., a questionnaire, a modified Nordic Musculoskeletal Questionnaire (mNMQ), and the Barthel activities of daily living (ADL) score. The details of each of these elements are discussed below.

The Survey Questionnaire

A 13-item questionnaire was developed to determine the musculoskeletal health as well as exercise and fitness activity-related status of the participants before and during the lockdown. Of the 13 questions, five evaluated the baseline activity status, four evaluated activity status during the lockdown, and four evaluated medical problems faced during the lockdown (Table 1).

**Table 1:** The survey questionnaire.

Question	Options
Prior to the lockdown (before March 22, 2020), how would you categorize your activity level	Community ambulatory (can go out of home independently or with minimal assistance)
	Independent home ambulatory (can ambulate at home independently or with minimal assistance)
	Dependent home ambulator (dependent on family/caregivers for ambulation)
	Bedbound
Prior to the lockdown, were you involved in any kind of health, wellbeing, and/or fitness-related activities	Yes
	No
If yes, prior to the lockdown, what kind of health, wellbeing, and fitness-related activities were performed by you	Yoga and meditation
	Walking/jogging
	Aerobic exercise other than walking/jogging
	Others (specify)
Prior to the lockdown, how much time did you devote to health, wellbeing, and fitness-related activities	Every day
	4–6 days/weeks
	2–3 days/week
	One day a week or less
If No, reason	Did not perform any activity due to time constraints
	Was not able to perform any activity due to physical/medical constraint
	Others (specify)
During the lockdown, were you involved in any sort of health, wellbeing, and fitness-related activities	Yes
	No
During the lockdown, what sort of health, wellbeing, and fitness-related activities were performed by you	Yoga and meditation
	Walking/jogging
	Aerobic exercise other than walking/jogging
	Others
During the lockdown, how much time did you devote daily for health, well-being, and fitness-related activities	Every day
	4–6 days/weeks
	2–3 days/week
	One day a week or less
If No, reasons	Did not perform any activity due to time constraints
	Was not able to perform any activity due to physical/medical constraints
	Others (specify)
During the lockdown, did your pre-existing musculoskeletal problems increase	Yes
	No
	NA
In case your pre-existing musculoskeletal problems increased, how did you manage it	Tele-consultation
	Self-management
	Continued with the existing treatment plan
	Did nothing
	Others
During the lockdown, has your weight	Increased
	Decreased
	Same as before
	Don’t know
Because of the lockdown, were the following medical services affected	Scheduled follow-up with orthopedic surgeons: Yes/No
	Scheduled surgery: Yes/No
	Scheduled physical therapy sessions: Yes/No
	Access to medicines/assistive devices/braces, etc., for your musculoskeletal problem: Yes/No
	Any other medical service that was affected: Yes/No
	Scheduled follow-up with orthopedic surgeons: Yes/No

The Modified Nordic Musculoskeletal Questionnaire

The original NMQ [6] evaluates musculoskeletal problems at two different time frames, viz., the past 12 months and the past 7 days. The time frames of the questionnaire were modified to compare the changes in musculoskeletal health before and after the lockdown. Consequently, the first part of the questionnaire was modified to reflect the baseline status of the participants, that is, 12 months prior to the start of the lockdown (March 22, 2020). The second part of the questionnaire was modified to cover changes in pre-existing problems during the lockdown (March 22 to June 8, 2020). The third part of the questionnaire was modified to cover new problems arising during the lockdown period (Table 2). No changes other than the time frames (for evaluating musculoskeletal problems) were made to the questionnaire.

**Table 2:** The modified Nordic Musculoskeletal Questionnaire.

Did you at any time during the past 12 months prior to March 22, 2020 had any trouble (such as pain, numbness, or discomfort)	During the lockdown, has the pre-existing trouble	Any new trouble during the lockdown
Neck (1) No (2) Yes	(1) Increased (2) Decreased (3) Remained the same	(1) No (2) Yes
Shoulder (1) No (2) Yes, right shoulder (3) Yes, left shoulder (4) Yes, both shoulders	(1) Increased (2) Decreased (3) Remained the same	(1) No (2) Yes, right shoulder (3) Yes, left shoulder (4) Yes, both shoulders
Elbow (1) No (2) Yes, right elbow (3) Yes, left elbow (4) Yes, both elbows	(1) Increased (2) Decreased (3) Remained the same	(1) No (2) Yes, right elbow (3) Yes, left elbow (4) Yes, both elbows
Wrist (1) No (2) Yes, right wrist (3) Yes, left wrist (4) Yes, both wrists	(1) Increased (2) Decreased (3) Remained the same	(1) No (2) Yes, right wrist (3) Yes, left wrist (4) Yes, both wrists
Hand (1) No (2) Yes, right hand (3) Yes, left hand (4) Yes, both hands	(1) Increased (2) Decreased (3) Remained the same	(1) No (2) Yes, right hand (3) Yes, left hand (4) Yes, both hands
Upper back (1) Yes (2) No	(1) Increased (2) Decreased (3) Remained the same	(1) Yes (2) No
Lower Back (1) Yes (2) No	(1) Increased (2) Decreased (3) Remained the same	(1) Yes (2) No
One or both hips/thighs/buttocks (1) Yes (2) No	(1) Increased (2) Decreased (3) Remained the same	(1) Yes (2) No
One or both knees (1) Yes (2) No	(1) Increased (2) Decreased (3) Remained the same	(1) Yes (2) No
One or both ankle/feet (1) Yes (2) No	(1) Increased (2) Decreased (3) Remained the same	(1) Yes (2) No

Barthel Activities of Daily Living Index

The Barthel ADL index [7] was used in its unmodified, original form. This is an ordinal scale that includes 10 items related to mobility and self-care. A higher score on the Barthel ADL index indicates better function.

Construction and validation of the research tool

A well-designed strategy was employed to construct and validate the research tool, the details of which are presented in the following sections.

Selection of Items for the Survey Instrument

A thorough literature review was conducted to identify problems associated with confinement among the elderly. Subsequently, a panel of five experts comprising orthopedic surgeons (n = 3) and community medicine physicians (n = 2) reviewed and shortlisted the survey questions.

Validation of the Survey Instrument

The face validity of the survey instrument was assessed by experts at the time of designing the questionnaire. Face validity is a subjective assessment of the questionnaire or score, usually by experts in the field [8]. Moreover, content validity was assessed by experts at the time of designing the questionnaire. Two external experts (i.e., those not involved in the design of the survey instrument) were asked to grade the relevance and clarity of survey items on a scale of 1 to 4 (1 = not clear/relevant, 2 = needs major revision, 3 = clear/relevant, but needs minor revision, 4 = clear/relevant). The content validity index (CVI) was determined for each item (I-CVI), as well as the overall survey instrument (S-CVI). The I-CVI was calculated by dividing the number of experts rating the item as 3 or 4 by the total number of experts. The S-CVI was calculated by determining the average of the I-CVI for all items [9]. Criterion validity of the mNMQ was ascertained after obtaining the survey results. Criterion validity is a measure of how well a score agrees with an established “gold standard” or concrete outcome measure [10]. For determining the criterion validity of the mNMQ score, respondents were asked if they experienced any worsening of their pre-existing musculoskeletal problems during lockdown (questionnaire item number 10); the response to this question was taken as the “gold standard.” Furthermore, the change in the net mNMQ score was compared between respondents who reported worsening of their problems versus those who did not. It was hypothesized that respondents who reported worsening of their musculoskeletal symptoms would have significantly worse (higher) net mNMQ scores than those who did not. The Mann-Whitney U test (the non-parametric equivalent of the independent t-test) was used to assess significant differences between the two groups. Two-tailed P-values were determined, and P-values of <0.05 were considered significant.

Survey administration

In a pilot analysis, the survey was administered to 10 volunteers over 60 years of age to determine whether the survey questions were easily understood and whether any modifications were required. Once this had been done, the survey was administered to eligible participants between June 19 and July 6, 2020, via a telephonic interview. A single researcher (RG) conducted all the interviews. Participants were informed about the study objectives and due consent was obtained before initiating the interview. A single interview lasted for approximately 10-15 minutes.

Outcome measures

**Figure 1:**
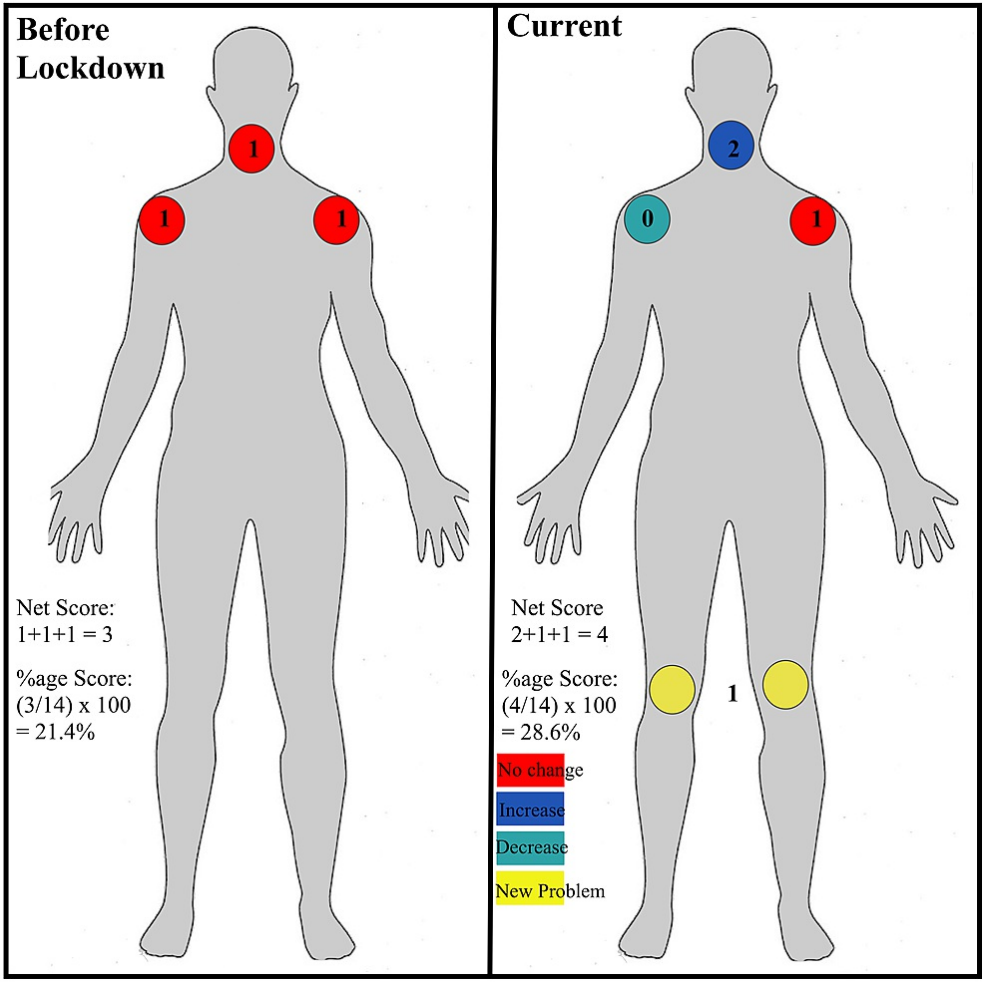
A representative example showing the calculation of the net modified Nordic Musculoskeletal Questionnaire score.

Outcome measures included: (a) reporting of all survey items; (b) change in the mNMQ; and (c) change in the Barthel ADL index. Previous studies have used the NMQ to assess region-wise distribution (percentage) of musculoskeletal problems in various patient cohorts [11]. Although this approach can identify anatomical regions with the highest percentage of musculoskeletal problems, it cannot be used to summarize and compare musculoskeletal problems in a single individual. To overcome this shortcoming, a system was developed to score the net NMQ in each patient. Each of the 14 anatomical regions (shoulders = 2, elbows = 2, wrists = 2, hands = 2, hips/thighs/buttock = 1, knees = 1, foot/ankle = 1, neck = 1, upper back = 1, and lower back = 1) included in the questionnaire were assigned a numerical score. For the first part (pre-lockdown problems), a score of 0 was assigned if the patient had no problem and 1 if the patient had a problem. The net pre-lockdown score was then divided by 14 (i.e., the total number of regions) and expressed as a percentage pre-lockdown score. To derive the score for the second part of the questionnaire, 1 was added to the pre-lockdown score if the problem was aggravated and 1 was subtracted from the pre-lockdown score if the problem improved. If the problem remained unchanged, it was scored the same as the pre-lockdown score. For the third part (new problems), a score of 0 was assigned if the patient had no problem, and 1 if the patient had a problem. The current net score was determined by calculating the total of the second and the third parts of the score. The net current score was divided by 14 to obtain the percentage current score (Figure 1). To facilitate subgroup analysis of the NMQ score, the 14 regions were divided on an anatomical basis into the upper limb, lower limb, and spine.

Statistical analysis

The normality of study data was ascertained using the Shapiro-Wilk test. Descriptive statistics and appropriate graphical measures were used to summarize the survey items. The Wilcoxon signed-rank test was used to compare the change in mNMQ score, as well as change in the Barthel ADL index. The Wilcoxon signed-rank test is analogous to the paired Student’s t-test and is used for samples that are non-normal in distribution. This test is used to compare two dependent samples, or repeated measurements on the same sample, by determining if there is a difference in their mean ranks [12]. Univariate analysis was conducted to determine which factors were associated with worsening (increase) in the net mNMQ score. Odds ratios (ORs) with 95% confidence intervals (CIs) were calculated for each variable of interest, and the overall significance was ascertained by the two-tailed Fisher’s exact test. In addition to the univariate analysis, multivariable logistic regression analysis was also performed to predict an increase in the net NMQ score from the variables included in the univariate analysis. Floor and ceiling effects were determined for the NMQ score by calculating the percentage of respondents with the lowest (ceiling effect) and the highest (floor effect) scores. In accordance with other orthopedic studies [13,14], if >15% of patients reported the lowest or the highest scores, it was considered significant. P-values of <0.05 were considered significant. Data analysis was performed using Microsoft Office Excel 2019 and Stata version 14.0 (StataCorp., College Station, TX, USA).

## Results

Validity of the survey instrument

The survey instrument was found to have acceptable face validity, as judged by a team of experts. Regarding clarity and relevance of the survey items, the I-CVI was found to be 1 for all survey items. The S-CVI was found to be 1. Regarding criterion validity, respondents who reported deterioration of their pre-existing musculoskeletal symptoms during lockdown were found to have a significantly worse (higher) net NMQ score compared to those who did not (z = -3.52, two-tailed P-value = 0.0004 ). On the basis of these observations, the survey instrument was found to be valid.

Response rate

**Figure 2:**
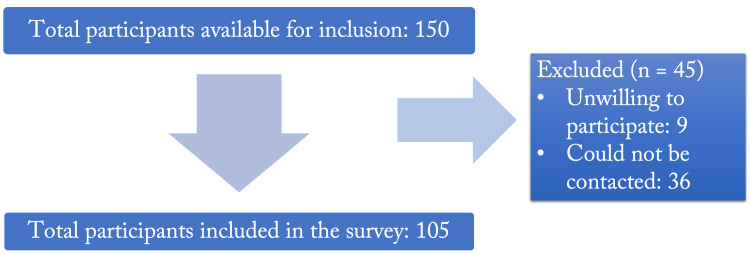
Flowchart of participants included in the survey.

Out of a total of 150 participants available for inclusion, 105 were recruited into this study; the response rate for the survey was therefore 70%. Of those who were not included, 36 could not be contacted and 9 refused to participate (Figure 2).

Sociodemographic profile of the survey respondents

The mean age of the survey respondents was 69.02 ± 7.57 years. The majority of the respondents were females (56.2%). Overall, 82% of the participants were educated, and 74% of the respondents were married. Hypertension (61%) and diabetes (31%) were the most common comorbidities among the study population. The vast majority (88.6%) of the participants were able to ambulate independently. The average follow-up of the study cohort at the time of conducting the survey was 10.3 ± 3.1 months (range = 4-17 months). Detailed sociodemographic information of the survey respondents is presented in Table 3.

**Table 3:** Sociodemographic profile of the survey respondents.

Variable	Frequency (%)
Age	
60–70 years	68 (64.8%)
71–80 years	28 (26.7%)
>80 years	9 (8.6%)
Gender	
Male	46 (43.8%)
Female	59 (56.2%)
Educational qualification	
Illiterate	19 (17.9%)
Literate and studied till fifth standard	8 (7.5%)
Sixth to seventh standard	29 (27.4%)
Eleventh standard to graduation	34 (32.1%)
Post-graduation and above	13 (14.2%)
Occupation	
Unemployed	2 (1.9%)
Employed	7 (6.7%)
Self-employed	8 (7.6%)
Homemaker	49 (46.7%)
Retired	39 (37.1%)
Marital status	
Married	74 (70.4%)
Widow (er)	31 (28.6%)
Living arrangements	
Living alone	5 (4.8%)
Only with spouse	19 (18.1%)
With spouse, adult children, and grandchildren	42 (40%)
With spouse and adult children	13 (12.4%)
With adult children and grandchildren	25 (23.8%)
With adult children only	1 (1%)
Comorbidities	
Hypertension	64 (61%)
Diabetes mellitus	33 (31%)
Cardiac ailments	10 (10%)
Osteoporosis	1 (1%)
Osteoarthritis	2 (2%)
Hyperthyroidism	15 (14%)
Level of mobility	
Community ambulator	93 (88.6%)
Independent home ambulator	11 (10.5%)
Dependent home ambulator	1 (1%)
Bed-bound	0 (0%)

Exercise and fitness-related activities before and during the lockdown

**Figure 3:**
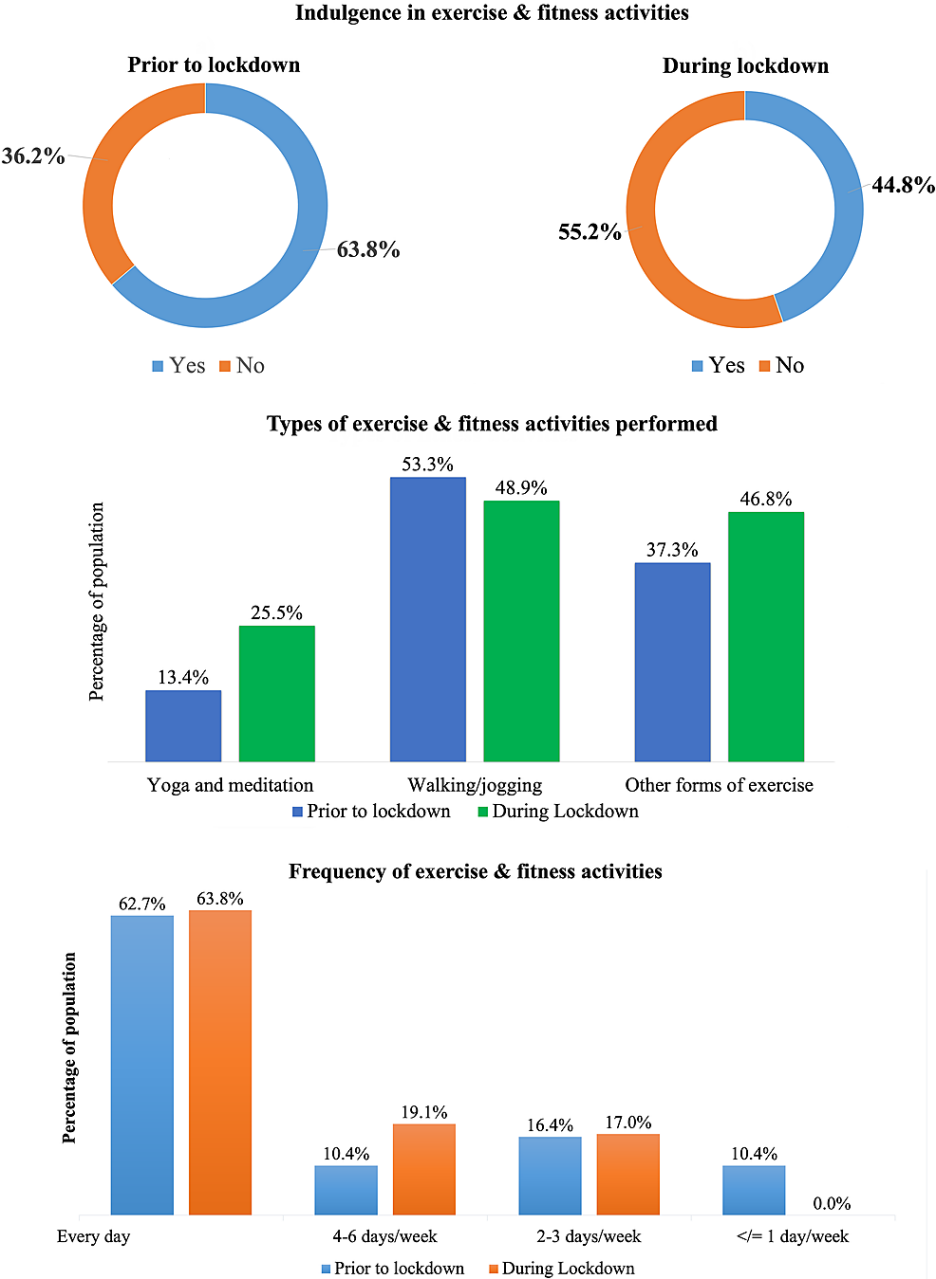
Exercise and physical activities performed by the respondents before and during the lockdown.

Overall, 63.8% (n = 67) of the survey respondents reported that they indulged in some kind of exercise or fitness-related activity before lockdown. However, this number decreased to 44.8% (n = 47) during the lockdown (Figure 3). This change was noted to be statistically significant (McNemar’s test: χ^2^ = 16.667; P < 0.001).

Before the lockdown, walking/jogging was the most common form of exercise performed by 53.3% of the survey respondents. However, this decreased to 48.9% during the lockdown. Yoga/meditation was performed by 13.4% of the survey respondents before the lockdown, which increased to 25.5% during the lockdown. The number of participants indulging in other forms of exercise such as cycling and low-impact aerobic exercises, stretching, and strengthening also increased from 37.3% before the lockdown to 46.8% during the lockdown (Figure 3).

The percentage of participants who exercised on a daily basis was almost similar in the pre-lockdown (62.7%) and lockdown (63.8%) periods. Similarly, the percentage of participants who exercised two to three days a week was also almost similar in the pre-lockdown (16.4%) and lockdown (17%) periods. However, the percentage of participants who exercised four to six days a week increased from 10.4% to 19.1% during the lockdown. On the other hand, the percentage of participants who exercised one day a week or less decreased from 10% to 0.065% (Figure 3).

Effect of the lockdown on medical services

Overall, 54.3% (n = 57) of the respondents reported problems with medical services during the lockdown. Cancellation of scheduled follow-up visits was the most frequent issue (24.8%), followed by cancellation of scheduled physical therapy sessions (10.5%). Cancellation of scheduled surgical procedures was reported by 4.8% of the respondents.

**Figure 4:**
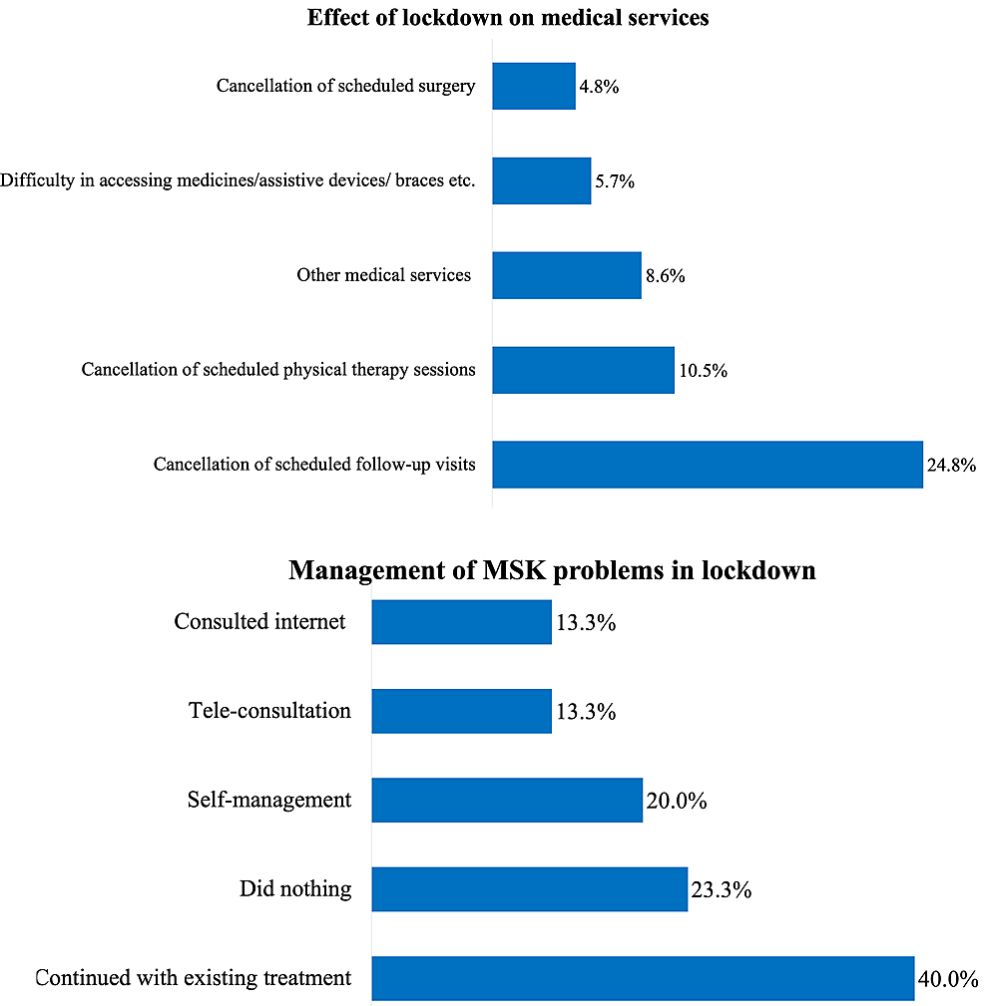
Effect of the lockdown on medical services, and management of musculoskeletal problems during the lockdown. MSK = musculoskeletal

Of those who experienced worsening of their existing musculoskeletal problems, 40% continued with their existing treatment plans during the lockdown. Self-management (in the form of massage, home remedies, etc.) was reported by 20% of the respondents. Only 13.3% of the respondents reported utilization of teleconsultation facilities (Figure 4).

Musculoskeletal problems before and during the lockdown

The majority of the respondents (48.7%, n = 63) had lower limb problems before the lockdown, of which knee joint problems were the most prevalent (37.3%, n = 46). Aggravation of pre-existing problems was most frequently noted in the lower limb (62.2%, n = 28), followed by the spine (28.8%, n = 13) and the upper limb (8.8%, n = 4). New-onset problems during the lockdown were noted most frequently in the lower limb (54.3%, n = 31), followed by the upper limb (26.3%, n = 15) and the spine (19.2%, n = 11) (Table 4).

**Table 4:** Prevalence of musculoskeletal symptoms in the respondents prior to and during the lockdown. Percentages are expressed as a factor of the total sites of involvement in each of the three columns. *The number of respondents who had problems at a particular site. Respondents with problems at multiple sites were accounted for at each site of involvement. Hence, the grand total reflects the total number of sites involved, rather than the total number of respondents.

Anatomical region		Pre-existing problems*	Aggravation of pre-existing problems*	New problems during the lockdown*
Upper limb	Shoulder	12 (9.7%)	3 (2.4%)	4 (7.0%)
	Elbow	1 (0.8%)	0 (0%)	3 (5.2%)
	Wrist	6 (4.8%)	1 (2.2%)	6 (10.5%)
	Hand	8 (6.5%)	0 (0%)	2 (3.5%)
	Total	27 (21.9%)	4 (8.8%)	15 (26.3%)
Lower limb	Hip/thigh/buttocks	3 (2.4%)	1 (2.2%)	3 (5.2%)
	Knee	46 (37.3%)	19 (42.2%)	18 (31.5%)
	Ankle/Feet	14 (11.3%)	8 (17.7%)	10 (17.5%)
	Total	63 (48.7%)	28 (62.2%)	31 (54.3%)
Spine	Neck	7 (5.7%)	1 (2.2%)	2 (3.5%)
	Upper back	4 (3.2%)	1 (2.2%)	4 (7.0%)
	Lower back	21 (17.0%)	11 (24.4%)	5 (8.7%)
	Total	33 (26.8%)	13 (28.8%)	11 (19.2%)
Grand total		123	45	57

Change in the modified Nordic Musculoskeletal Questionnaire score and Barthel activities of daily living index

The net mNMQ score increased from 7.96% before the lockdown to 13.12% at the time of the survey, and this difference was statistically significant (P = 0.000). A statistically significant increase in the mNMQ score was noted for the lower limb (P = 0.000) and the spine (P = 0.002), but not for the upper limb (P = 0.052) (Table 5).

The Barthel ADL index showed a small increase from 18.27 ± 1.94 prior to the lockdown to 18.43 ± 1.88 at the time of the survey. This difference was not statistically significant (P = 0.467) (Table 5).

**Table 5:** Change in the mNMQ score and the Barthel ADL index mNMQ = modified Nordic Musculoskeletal Questionnaire; ADL = activities of daily living

Score	Before lockdown (Mean ± SD)	Current (Mean ± SD)	P-value
mNMQ score (%)			
Upper limb	1.56 ± 3.83	2.24 ± 5.71	0.052
Lower limb	4.29 ± 5.20	7.62 ± 11.35	<0.0001
Spine	2.11 ± 3.83	3.27 ± 5.61	0.002
Net	7.96 ± 9.41	13.12 ± 17.72	<0.0001
Barthel ADL index	18.27 ± 1.94	18.43 ± 1.88	0.467

Factors influencing an increase in the modified Nordic Musculoskeletal Questionnaire score

Respondents whose medical services were affected during the lockdown had a significantly greater increase (worsening) in the net mNMQ score, in contrast to those whose medical services were not affected (OR = 6.16, 95% CI = 2.51-15.08; P = 0.0001). On the other hand, age, gender, ambulatory status, increase in body weight, and indulgence in exercise before and during lockdown had no impact on the change in the net mNMQ score (Table 6).

**Table 6:** Factors influencing worsening (increase) in the net modified Nordic Musculoskeletal Questionnaire score (univariate analysis). *Determined by Fischer’s exact test, two-tailed P-values have been reported. Values in italics indicate a significant result (P < 0.05). OR = odds ratio; CI = confidence interval

Factor	Categories	OR (95% CI)	P-value*
Age	(a) >70 years (b) <70 years	0.54 (0.21–1.35)	0.21
Ambulatory status	(a) Community ambulator (b) Home ambulator	0.41 (0.1–1.65)	0.21
Exercise or fitness activities during lockdown	(a) Yes (b) No	0.94 (0.40–2.23)	0.78
Exercise or fitness activities prior to lockdown	(a) Yes (b) No	0.82 (0.34–2.00)	0.68
Gender	(a) Male (b) Female	0.51 (0.21–1.22)	0.11
Weight gain during lockdown	(a) Yes (b) No	1.44 (0.42–4.81)	0.58
Whether any medical services were affected	(a) Yes (b) No	6.16 (2.51–15.08)	0.0001

Results of multivariable logistic regression revealed that after controlling for other variables (age, gender, ambulatory status, physical activity before and during lockdown, weight increase during lockdown), respondents whose medical services were impacted during lockdown had significant worsening (P = 0.001) of the mNMQ score (Table 7).

**Table 7:** Multivariable logistic regression analysis of factors influencing worsening (increase) in the net modified Nordic Musculoskeletal Questionnaire score. OR = odds ratio; SE = standard error; CI = confidence interval

Factor	OR	SE	z	P-value	95% CI
Age	0.41	0.28	−1.29	0.198	0.01–1.59
Ambulatory status	0.28	0.30	−1.20	0.231	0.03–2.27
Exercise or fitness activities during the lockdown	1.60	1.25	0.61	0.543	0.35–7.36
Exercise or fitness activities before the lockdown	1.17	0.96	0.19	0.852	0.23–5.84
Gender	1.11	0.76	0.15	0.879	0.29–4.26
Weight increase during the lockdown	1.61	1.09	0.71	0.478	0.43–6.07
Whether any medical services were affected	10.54	7.44	3.33	0.001	2.64–42.07

Ceiling and floor effects of the modified Nordic Musculoskeletal Questionnaire score

Overall, 35% of respondents reported the best possible NMQ score (0) prior to lockdown, whereas 33% of the respondents reported the best possible score at the time of the survey. Hence, a significant ceiling effect was deemed to be present. However, no floor effect was noted, with only 1% of the survey participants reporting the worst (highest) NMQ scores, both before the lockdown and at the time of the survey.

## Discussion

Continued mobility is of paramount importance among the elderly. Physical inactivity has been shown to adversely impact cardiovascular function, cognition, and musculoskeletal health, resulting in increased mortality in this population [15,16]. The degree of confinement imposed on the elderly due to strict lockdown measures implemented during the peak of the COVID-19 pandemic in India was unprecedented. However, the effect of such confinement on the musculoskeletal health of the geriatric population has been sparsely reported in the literature.

Brady et al. [17] conducted an online survey to determine the effect of COVID-19 lockdowns on the musculoskeletal health of 345 adult patients with rheumatoid arthritis in the United Kingdom. Participants were evaluated using the National Institutes of Health-American Association of Retired Persons Diet and Health Study Questionnaire, International Physical Activity Questionnaire-Short Form, McGill Pain Questionnaire and Visual Analogue Scale, Multidimensional Fatigue Inventory, Hospital Anxiety and Depression Scale, and the Subjective Vitality Scale. Using hierarchical linear regression analysis, they demonstrated a positive effect of physical activity on mental health and overall wellbeing.

To determine the effect of lockdown on the physical activity status of the geriatric population, Pérez et al. [18] conducted a telephonic questionnaire-based study among 98 frail Spanish elders. Physical activity was evaluated using the Brief Physical Activity Assessment Tool. The authors noted less-than-sufficient physical activity in approximately one-third of the participants. Depressive symptoms, fatigue, and living alone decreased the odds of maintaining adequate physical activity, whereas reading and maintaining social networks increased it.

To our knowledge, this is the first study from the Indian subcontinent evaluating the effects of lockdown on the musculoskeletal health of the urban geriatric population. In this study, we made several interesting observations. First, we observed that the physical activity status of the geriatric population declined significantly during the lockdown, which is in concurrence with that of Pérez et al., who showed a similar decline in the elderly Spanish population [18]. We also observed that the lockdown impacted the delivery of routine medical services and that many elderly individuals did not have access to routine outpatient and physical therapy services during this period. Importantly, we noted that the overall musculoskeletal health, as determined by the net mNMQ score, deteriorated during the lockdown. This was attributable to worsening of the lower limb and spinal problems. Regular physical activity is advocated as one of the key components of the management of musculoskeletal problems. Hence, we explored whether those elders who indulged in exercise and physical activity before and during the lockdown had less worsening of their musculoskeletal symptoms. However, we noted that these parameters were not significantly associated with worsening of the net mNMQ scores. On the other hand, those elders whose medical services were affected during lockdown had significantly worse scores. This highlights the importance of continued access to medical services for the elderly during periods of confinement.

This study has several strengths. Although the survey itself was cross-sectional, it was performed on a prospective cohort of patients who continue to be under follow-up. The study was performed in strict accordance with SURGE [5], and due diligence was followed in designing, piloting, and validating the survey instrument. We have also described a novel method to score the NMQ, and we believe that this may be useful to other researchers. Notwithstanding the strengths, there are a few limitations of this study that need to be mentioned. First, the sample size was relatively small; of the 150 participants available for inclusion, only 105 chose to participate in the survey. Therefore, the results should be extrapolated with caution. We acknowledge that our finding of no association of physical activity with musculoskeletal problems may be attributable to a type II error owing to the small sample size. We also acknowledge the inherent limitation of the NMQ. The questionnaire is designed to give higher weightage to upper limb problems, whereas hips, knees, and ankles are considered a single region. This can lead to an under-representation of lower limb problems when determining the overall score. We did not alter this aspect of the questionnaire as it has been extensively validated in the literature, and major changes could have potentially compromised the structure of the questionnaire. The under-representation of lower limb problems notwithstanding, we could demonstrate significant worsening of lower limb problems in our participants. Finally, we noted a significant ceiling effect of the mNMQ score. This implies that the ability of the score to discriminate between those with mild to no musculoskeletal symptoms may be questionable. Hence, future studies should explore approaches to improve this aspect of the score.

## Conclusions

We have demonstrated that the COVID-19 lockdown resulted in a significant decline in the physical activity status of the elderly, as well as worsening of musculoskeletal problems, especially in the lower limbs and spine. If such drastic measures need to be implemented in the future for any reason, policymakers should ensure uninterrupted access to medical services and develop optimal home-based physical activity regimens to counter problems associated with sedentarism among the elderly.
